# Pleuropneumonectomy for a large thymoma with multiple pleural dissemination using median sternotomy followed by posterolateral thoracotomy

**DOI:** 10.1186/s40792-015-0071-z

**Published:** 2015-09-02

**Authors:** Yasushi Shintani, Ryu Kanzaki, Hidenori Kusumoto, Tomoyuki Nakagiri, Masayoshi Inoue, Meinoshin Okumura

**Affiliations:** Department of General Thoracic Surgery, Osaka University Graduate School of Medicine, 2-2-L5 Yamadaoka, Suita City, Osaka 565-0871 Japan

**Keywords:** Thymoma, Pleuropneumonectomy, Pleural dissemination, Multimodal treatment

## Abstract

We present 2 cases of a large thymoma with invasion to the hilum of the lung and pleural dissemination. Case 1: a 47-year-old woman was diagnosed with a type B3 thymoma with abundant left pleural effusion and multiple pleural masses, Masaoka stage IVa. A radical resection was planned after chemical pleurodesis and systemic chemotherapy. The left main pulmonary artery and left upper and inferior veins were dissected and resected in the pericardium, while the left main bronchus was cut behind the pericardium through a median sternotomy. Next, the median incision was closed and a left posterolateral thoracotomy was made, thus allowing the pleuropneumonectomy to be safely performed. Case 2: a 47-year-old woman was diagnosed with a type B3 thymoma with lymph node swelling and multiple pleural masses, indicating Masaoka stage IVb. Following induction chemotherapy, a thymothymectomy combined with a right pleuropneumonectomy was performed under a median sternotomy followed by a right posterolateral thoracotomy. The left brachiocephalic vein (BCV) was reconstructed with a ringed polytetrafluoroethylene (PTFE) graft, followed by resection of the right BCV. Next, the right main pulmonary artery and right upper and inferior veins were resected in the pericardium, and the right main bronchus was cut behind the pericardium, followed by reconstruction of the right BCV. Finally, the median incision was closed and a right posterolateral thoracotomy was made, thus allowing performance of a safe pleuropneumonectomy. The median sternotomy allowed safe dissection of pulmonary vessels surrounding the hilum of the lung and, in combination with a posterolateral thoracotomy, was required for performing a pleuropneumonectomy in patients with a huge thymoma with pleural dissemination.

**Electronic supplementary material:**

The online version of this article (doi:10.1186/s40792-015-0071-z) contains supplementary material, which is available to authorized users.

## Background

A Masaoka stage IV thymoma is defined as a tumor with pleura or pericardial dissemination, while standard treatment for affected patients has not been established [1]. Here, we report two patients with a stage IV thymoma successfully treated with chemotherapy followed by a radical resection with a pleuropneumonectomy through anterior approach combined with posterolateral thoracotomy.

## Case presentation

### Case 1

**Fig. 1:**
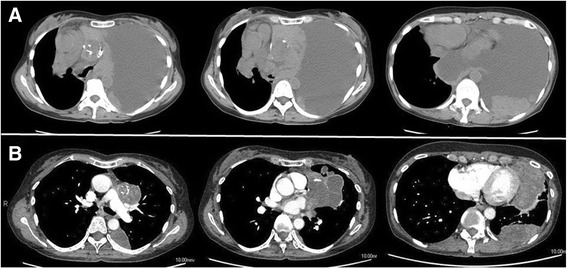
**a** Chest CT scan showing an anterior mediastinal tumor with abundant left pleural effusion and multiple pleural tumors. **b** CT scan image obtained after chemical pleurodesis and systemic chemotherapy showing a mediastinal tumor that has invaded the hilum of the left lung and multiple disseminated masses

A 47-year-old woman was presented with pain in the dorsal region, accompanied by dyspnea. Chest X-ray findings showed abundant left pleural effusion and a computer tomography (CT) scan revealed a mediastinal tumor sized 13 × 12 × 9.6 cm, abundant left pleural effusion, and pleural tumor, Masaoka stage IVa (Fig. 1a). Histopathologic analysis of a CT-guided percutaneous core biopsy specimen resulted in a diagnosis of type B3 thymoma. Chemical pleurodesis with 5 Klinische Einheit (KE) of OK-432 was performed, and systemic chemotherapy with cisplatin (60 mg/m^2^ on day 1) and etoposide (120 mg/m^2^ on days 1–3) were administered over 4 cycles with partial response. Subsequently, CT scanning demonstrated a mediastinal tumor invading the upper lobe of the left lung, while multiple disseminated masses were also apparent throughout the pleural cavity because of an absence of pleural fluid (Fig. 1b). Following a thorough clinical assessment, a radical resection with a left pleuropneumonectomy was proposed. The anterior mediastinal tumor was considered to have invaded the hilum of the left lung including the main pulmonary artery; we planned to dissect the hilum in the pericardium through anterior approach. After a median sternotomy, we found that the mediastinal mass involved most of the pericardium and the hilum of the left lung. The left main pulmonary artery and left upper and inferior veins in the pericardium were resected. We considered it difficult to excise the left main bronchus through a left posterolateral thoracotomy because the tumor invaded the hilum of the lung; thus, the left main bronchus was cut behind the pericardium through a median sternotomy (Additional file 1: Video 1). Next, the median incision was closed and a left posterolateral thoracotomy at the fifth intercostal incision was performed for a pleuropneumonectomy (Additional file 2: Video 2). The pericardium and diaphragm were reconstructed with a Gore-Tex patch. Surgical operating time was 617 min, and total blood loss was 3650 cc. The postoperative course was uneventful, and the patient was discharged on postoperative day 26. Four years after the pleuropneumonectomy procedure, a chest CT scan revealed no disease.

### Case 2

**Fig. 2:**
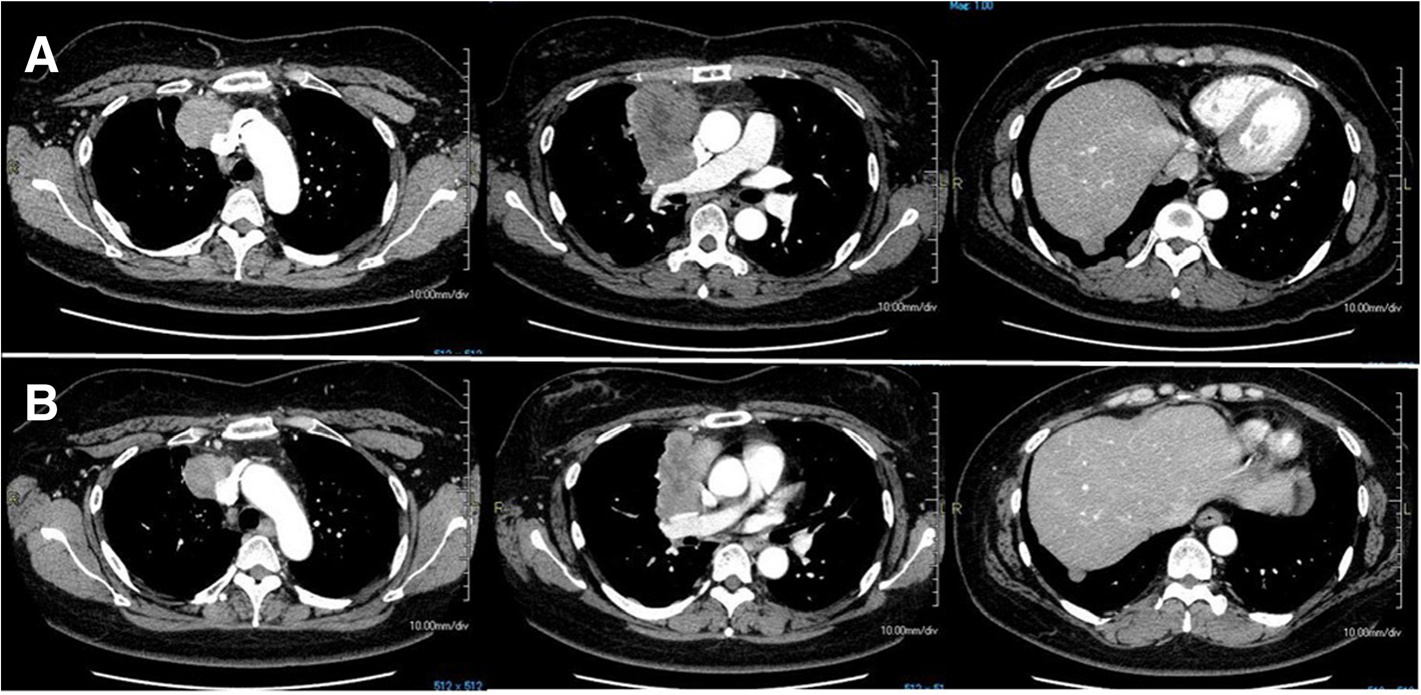
**a** Chest CT scan showing an anterior mediastinal tumor that is tightly adherent to the superior vena cava (SVC) and pulmonary trunk with swelling of the tracheobronchial lymph nodes and multiple pleural masses. **b** Chest CT scan showing a regression of the mediastinal and pleural masses after induction chemotherapy

**Fig. 3:**
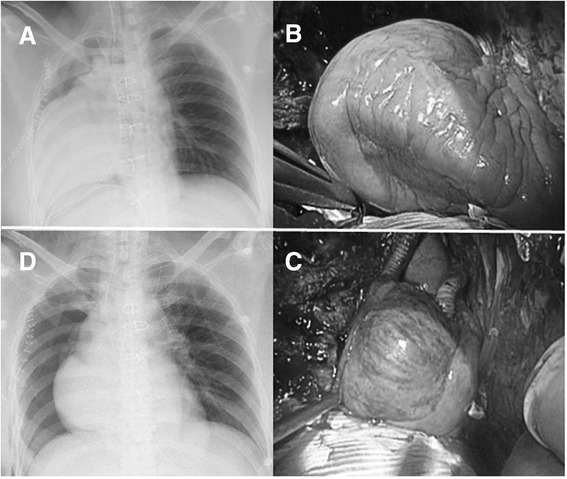
**a** Chest X-ray showing protrusion of the heart into the right thorax. **b** Intraoperative view showing cardiac herniation from the repaired pericardial defects. **c** Intraoperative view showing the heart returned to its normal position. **d** Chest X-ray showing that the heart was returned to its normal position after re-thoracotomy

A 47-year-old woman was presented with cough and dyspnea. Chest X-ray findings showed mediastinal enlargement, and CT scan images revealed a mediastinal tumor sized 12 × 10 × 8.5 cm, which was tightly adhered to the superior vena cava (SVC) and pulmonary trunk, along with swelling of the tracheobronchial lymph nodes and multiple pleural masses, Masaoka stage IVb (Fig. 2a). A CT-guided percutaneous core biopsy procedure resulted in a diagnosis of type B2 thymoma. Following induction chemotherapy (every 3 weeks for 3 cycles) with doxorubicin (40 mg/m^2^ on day 1), cisplatin (50 mg/m^2^ on day 1), vincristine (0.6 mg/m^2^ on day 3), and cyclophosphamide (700 mg/m^2^ on day 4), a second chest CT scan examination showed regression of the mediastinal and pleural masses (Fig. 2b). We planned a thymothymectomy combined with a right pleuropneumonectomy under a median sternotomy, which allowed us to approach the great vessels in the pericardium. To control the right pulmonary artery, the left brachiocephalic vein (BCV) was reconstructed with a ringed polytetrafluoroethylene (PTFE) graft, followed by resection of the right BCV. Next, the right main pulmonary artery and right main bronchus were cut, followed by reconstruction of the right BCV (Additional file 3: Video 3). We then performed a right posterolateral thoracotomy at the fifth intercostal incision for a pleuropneumonectomy. The pericardium and diaphragm were reconstructed with a GORE-TEX patch. Surgical time was 811 min and total blood loss was 2700 cc. On postoperative day 1, chest X-ray findings revealed protrusion of the heart into the right thorax (Fig. 3a) and a re-thoracotomy was performed, during which we found cardiac herniation from the repaired pericardial defects (Fig. 3b). The heart was returned to its normal position (Fig. 3c), and the pericardial defect was repaired again with a GORE-TEX patch. The patient recovered (Fig. 3d) during a postoperative course that was uneventful and discharged on postoperative day 35. One year later, chest PET-CT scanning found a paratracheal and pleural hypermetabolic spots indicating recurrence in the thoracic cavity, and the masses were treated by radiotherapy. At the time of writing, the patient was alive with recurrent disease for 4 years.

### Comments

Optimal treatment for a Masaoka stage IVa thymoma with pleural dissemination has not been established. Even in patients with a locally advanced and initially unresectable thymoma, treatments with induction chemotherapy followed by resection have resulted in good overall survival rates [2, 3]. Kondo et al. also reported that thymomas have a moderate response rate to chemotherapy or radiotherapy; thus, multimodality therapy involving surgery, chemotherapy, and radiotherapy appears to increase the rate of complete resection and survival in the advanced thymomas [4]. Some have reported that surgical debulking is acceptable for an invasive thymoma, because of the potential for a favorable outcome [5]. We agree with that concept and prefer conservative treatment with resection of visible disseminated nodules by a partial pleurectomy for patients with a stage IVa thymoma. Whereas macroscopic total resection of tumors appears to be a promising prognostic factor in Masaoka stage IVa thymomas, it was impossible to conserve the lung because of tumor invasion to the hilum especially to the main pulmonary artery in both cases; thus, we considered that a pleuropneumonectomy was a feasible approach in these present cases. Several reports of a pleuropneumonectomy procedure for stage IVa thymoma have demonstrated it to be technically feasible and showed short-term good results [6, 7]. A pleuropneumonectomy would seem to be warranted for a small number of highly selected patients with advanced or recurrent thymoma extensively involving the pleural space. Thymoma is a malignancy with a generally good prognosis [8]; thus, further evaluation of the long-term outcomes is needed to determine the indications for pleuropneumonectomy for patients with advanced thymoma.

Generally, the intrathoracic vessels and main bronchus are dissected using a thoracotomy during a pleuropneumonectomy for patients with a malignant mesothelioma. However, in that procedure for patients with a thymoma, pulmonary vessels cannot be dissected because of tumor invasion to the hilum of the lung. Furthermore, a median sternotomy or posterolateral thoracotomy alone is not adequate for pleural exploration. Although the combination of a partial median sternotomy with an anterior thoracotomy (hemi-clamshell) facilitates exposure of the pleural cavity, this approach is not adequate for pleuropneumonectomy. Thus, we dissected pulmonary vessels in the pericardium using a median incision in the present cases. Yang et al. reported that a median sternotomy was added for en bloc total thymectomy immediately after resecting the lungs and pleura via a posterolateral thoracotomy [9]. We prefer to secure pulmonary great vessels before pulmonary resection to avoid massive bleeding from lung parenchyma; thus, we first dissected pulmonary vessels in the pericardium using a median incision. In addition, the main bronchus was easily excised and cut within the main bronchus about 2 cm below the carina through a median sternotomy. A short mainstem bronchial stump is optimal when performing pneumonectomy and pleuropneumonectomy; thus, this approach may be useful for these patients. Thereafter, the median incision was closed and a posterolateral thoracotomy at the fifth intercostal incision was made following a position change from a spine to lateral decubitus position, thus allowing the pleuropneumonectomy to be safely performed. Our approach may result in a safe procedure for resection of a large thymoma invading the hilum of the lung with pleural dissemination. Our second case developed cardiac herniation and underwent a re-thoracotomy for repair. Defects in the pericardium are larger with a thymothymectomy combined with a pleuropneumonectomy using median and posterolateral incisions as compared to a pleuropneumonectomy using a posterolateral incision; thus, pericardial defects should be carefully repaired with a strong material.

## Conclusions

We treated two patients with a large thymoma with invasion to the hilum of the lung and pleural dissemination. The median sternotomy allowed safe dissection of pulmonary vessels surrounding the hilum of the lung and, in combination with a posterolateral thoracotomy, was required for performing a pleuropneumonectomy in patients with a huge thymoma with pleural dissemination.

## Consent

Written informed consent was obtained from the patient for publication of this case report and any accompanying images.

## Additional files

Additional file 1:
**Video 1.** After performing a median sternotomy, the left main pulmonary artery and left upper and inferior veins were resected in the pericardium, and the left main bronchus was cut behind the pericardium through a median sternotomy. Additional file 2:
**Video 2.** A left posterolateral thoracotomy at the fifth intercostal incision was performed for a pleuropneumonectomy. The pericardium was reconstructed with a GORE-TEX patch. Additional file 3:
**Video 3.** After reconstruction of the left brachiocephalic vein (BCV) with a polytetrafluoroethylene (PTFE) graft and resection of the right BCV, the right main pulmonary artery and bronchus were resected by a stapler, followed by reconstruction of the right BCV with a PTFE graft. Then, a right posterolateral thoracotomy at the fifth intercostal incision was performed for a pleuropneumonectomy.
